# Advanced bioengineering strategies broaden the therapeutic landscape for corneal failure

**DOI:** 10.3389/fbioe.2024.1480772

**Published:** 2024-11-13

**Authors:** Reem Al Monla, Vincent Daien, Frederic Michon

**Affiliations:** ^1^ Institute for Neurosciences of Montpellier, INSERM, University of Montpellier, Montpellier, France; ^2^ Department of Ophthalmology, Gui de Chauliac Hospital, Montpellier, France; ^3^ Sydney Medical School, The Save Sight Institute, The University of Sydney, Sydney, NSW, Australia

**Keywords:** bioartificial cornea, tissue engineering, cornea, corneal pathologies, 3D bioprinting, hydrogel scaffolds

## Abstract

The cornea acts as the eye foremost protective layer and is essential for its focusing power. Corneal blindness may arise from physical trauma or conditions like dystrophies, keratitis, keratoconus, or ulceration. While conventional treatments involve medical therapies and donor allografts—sometimes supplemented with keratoprostheses—these options are not suitable for all corneal defects. Consequently, the development of bioartificial corneal tissue has emerged as a critical research area, aiming to address the global shortage of human cornea donors. Bioengineered corneas hold considerable promise as substitutes, with the potential to replace either specific layers or the entire thickness of damaged corneas. This review first delves into the structural anatomy of the human cornea, identifying key attributes necessary for successful corneal tissue bioengineering. It then examines various corneal pathologies, current treatments, and their limitations. Finally, the review outlines the primary approaches in corneal tissue engineering, exploring cell-free, cell-based, and scaffold-based options as three emerging strategies to address corneal failure.

## 1 Introduction

The cornea is a convex, transparent layer that covers the pupil, iris, and anterior chamber of the eye (Duke-Elder, 1958). Its primary role is to protect the underlying ocular structures, transmit light, and provide approximately two-thirds of the refractive power of the eye by focusing light onto the retina with minimal optical loss or scattering. Structurally, the cornea is composed of five distinct layers, and damage to any one of these can result in vision impairment (Maurice, 1957; Laibson, 1972).

Globally, over 10 million people are affected by corneal blindness due to various injuries or diseases. The most common treatment for vision restoration in such cases is corneal transplantation, or keratoplasty (KP), either partially or fully replacing the damaged cornea. However, KP faces significant challenges, including a shortage of donor corneas and the risk of immune rejection (Paton, 1954; Mannis and Krachmer, 1981; Aquavella, 2007). These challenges underscore the urgent need for innovative alternative solutions, such as bioengineered corneas, to replace native corneal tissue (Germain et al., 2000; Carlsson et al., 2003; Fagerholm et al., 2009).

## 2 Anatomy of the cornea

### 2.1 General description of the cornea

**FIGURE 1:**
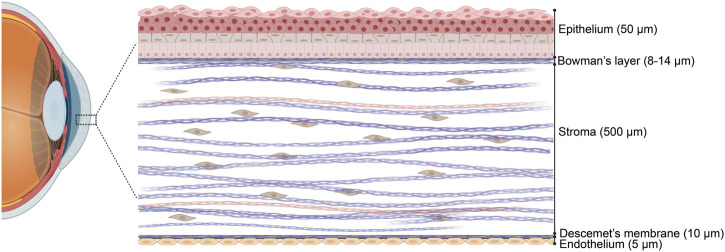
Schematic representation of a human cornea cross section in a hierarchical arrangement. Three cellular layers, namely, the epithelium, stroma, and endothelium, and two acellular basement membranes, namely, Bowman’s layer and Descemet’s membrane. Figure designed with Biorender (https://app.biorender.com).

The adult human cornea, measuring approximately 550 µm in thickness, is divided into three main regions and has an oval shape, with vertical dimensions of 10–11 mm and horizontal dimensions of 11–12 mm (Ehlers et al., 1975; Doughty and Zaman, 2000; Piñero et al., 2008). Corneal thickness decreases with age and tapers from the periphery toward the center. The outermost layer is the epithelium, separated from the stroma by Bowman’s layer (BL), which is 8–12 µm thick. The middle section, composed of the stroma, accounts for the majority of the cornea’s thickness. Descemet’s membrane (DM) serves as the basement membrane between the stroma and the corneal endothelium (Figure 1) (Li et al., 1997; Hayashi et al., 2002; Reinstein et al., 2008). Each layer of the cornea features a distinct extracellular matrix (ECM) composition, with varying types of collagen fibers, as detailed in Table 1.

**TABLE 1:** Variations in ECM composition and collagen types across the different layers of the human adult cornea.

Cornea layer	ECM composition	Collagen type	Reference
Epithelium	Hyaluronoan synthases Agrin Syndecan-1 Exotosin Glycosyl transferase1 enzymes Lubricin Aggrecan Versican	II, IX	Michelacci (2003), Puri et al. (2020)
Bowman’s layer	Thrombospondins 1 Perlecan	IV, VII, XVIII	Michelacci (2003), Espana and Birk (2020)
Stroma	Fibronectin proteoglycan Glycosaminoglycan Small leucine-rich proteoglycans (*Decorin, Lumican, Biglycan, Keratocan, Fibromodulin, Osteoglycin)*	I (75% of total) V, VI (17%), XII, XIII, XIV, V-I interaction	Michelacci (2003), Espana and Birk (2020), Puri et al. (2020)
Descement’s membrane	Thrombospondins 1, Fibronectin, Laminin, Heparan sulphate, Dermatan sulphate, Keratan sulphate	IV, VIII, XII	Michelacci (2003), Espana and Birk (2020), Parekh et al. (2021)
Endothelium	Fibronectin, Vitronectin, Fibrillins, Periostin, Tubulin β-1 and 4a chains	XII	Michelacci (2003), Parekh et al. (2021)

Even microscale deviations in the anterior corneal surface can result in significant changes to refractive power and visual function. The cornea’s light transmission efficiency ranges from 80% to 98% across wavelengths of 450–1,000 nm (Beems and Van Best, 1990). These high levels of light transmission in the visible spectrum (380–750 nm) are achieved despite the cornea’s dense collagen structure, a result of the highly organized packing and nanoscale architecture of the collagen fibers, which maintain transparency while allowing light to pass through (Maurice, 1957; Tseng et al., 2022).

Anatomically, the cornea can be divided into central and peripheral regions, each with unique structural and physiological characteristics. The central cornea is thinner but five times more innervated than the periphery (He et al., 2010; Bergmanson et al., 2019). From a mechanical perspective, the peripheral cornea is stiffer and displays a nonlinear stress-strain response, stiffening further under elevated strains. The elastic strength of the cornea, measured by Young’s modulus of elasticity, ranges from 82 to 530 kPa for the anterior cornea and from 28 to 162 kPa for the posterior stroma (Dias et al., 2013).

A thorough understanding of the architecture, cellular functions, and composition of each corneal layer is essential for successful tissue engineering or bioprinting efforts, providing a crucial foundation for experimental design and 3D modeling.

### 2.2 Corneal epithelium

The corneal epithelium originates from the surface ectoderm around the 5th to 6th week of embryonic development (Wulle and Richter, 1978). This outermost layer of the cornea, measuring 40–50 µm thick, is composed of 4–6 layers of nonsecretory stratified epithelial cells and is protected by a thin tear film that serves as a vital barrier against the external environment 20 three main types: basal cells forming the posterior layer, wing (or suprabasal) cells, and flat polygonal superficial cells with apical microvilli (Pellegrini et al., 1999; Delmonte and Kim, 2011; Lobo et al., 2016; Sridhar, 2018).

The maintenance of the corneal epithelium is explained by the X, Y, Z hypothesis proposed by Thoft and Friend, which describes a balance among three processes: basal epithelial cell proliferation (X), centripetal migration of peripheral cells (Y), and the loss of superficial epithelial cells (Z). This equilibrium ensures the consistent renewal of the corneal epithelium (Thoft and Friend, 1983).

The peripheral cells (Y) are known as limbal epithelial stem cell (LESCs), which reside exclusively in the limbal zone and are essential for the long-term maintenance and regeneration of the corneal epithelium after injury (Huang and Tseng, 1991). The heterogeneous limbal niche, located in the basal epithelial layer and within the palisades of Vogt, is rich in LESCs (Dua et al., 2005). LESCs maintain a steady cell population by proliferating and differentiating into transit amplifying cells (TACs), which then progressively migrate into the cornea to eventually differentiate into corneal epithelial cells (CECs) (Yoon et al., 2014).

### 2.3 Bowman’s layer

Bowman’s Layer (BL) is acellular and consists primarily of proteoglycans and randomly oriented collagen fibrils, predominantly type I, though types III, V, and XII are also present (Jacobsen et al., 1984; Marshall et al., 1991; Hayashi et al., 2002; Merindano et al., 2002). While several hypotheses exist regarding the function of BL, some remain controversial. For instance, Tong et al. proposed that BL plays a crucial mechanical role in maintaining corneal shape; however, this was challenged by studies indicating that BL is not essential for corneal stiffness (Tong et al., 2019; Torres-Netto et al., 2021).

Additionally, BL may serve as a barrier regulating the exchange of growth factors between the epithelium and stroma (Gordon et al., 1994; Mohan et al., 1997; Torricelli et al., 2015). This barrier function could have important implications for corneal homeostasis and wound healing, although further research is needed to fully understand these mechanisms.

### 2.4 Corneal stroma

The human corneal stroma forms the central core of the cornea, accounting for approximately 80%–85% of its total thickness. The unique structure and composition of the extracellular matrix (ECM) in the stroma are essential for maintaining light transmittance. Subtle variations in fibrillar diameter and spacing exist between the anterior and posterior stroma. In the anterior stroma, fibrils are more tightly packed, and the anterior and mid-stromal lamellae are heavily interwoven. In contrast, the posterior lamellae are less interwoven and become increasingly hydrated toward the central cornea (Meek and Knupp, 2015).

The transparency of the corneal stroma is largely attributed to the precise spatial arrangement of its narrow, densely packed collagen fibrils. The refractive index of these collagen fibrils closely matches that of the surrounding ground substance, resulting in minimal light scattering due to the small diameter of the fibrils—significantly smaller than the wavelength of visible light (Bron, 2001). This concept was initially proposed by Maurice, who demonstrated that the spatial organization of stromal collagen fibrils, oriented in specific directions, facilitates the forward propagation of secondary radiation. Known as the hexagonal crystalline lattice theory, this hypothesis emphasized the importance of fibril arrangement for corneal transparency (Maurice, 1957).

However, later studies questioned the existence of a long-range ordered crystalline lattice, instead describing the collagen fibril arrangement as a “short-range ordered” structure that enables optical interference effects (Farrell and Hart, 1969; Sayers et al., 1982; Worthington and Inouye, 1985). More recent research by Tseng et al. suggests that corneal transparency is influenced more by fibril diameter than by geometric arrangement into hexagonal lattices. According to their findings, the optical penetration of specific wavelengths is determined by the polydispersity of collagen fibrils rather than their spacing (Tseng et al., 2022).

The maintenance of this fibrillar arrangement is supported by collagen fibril-associated proteoglycans, which, along with matrix metalloproteinases and collagen, contribute to the cornea’s mechanical strength and transparency (Cheng and Pinsky, 2013). Type I collagen is the predominant collagen type in the corneal stroma, with smaller amounts of types V, VI, XII, XIII, XIV, and XXIV. The presence of type III collagen in the normal corneal stroma remains a subject of debate (Ihanamäki et al., 2004).

These collagen fibrils are heterotypic, comprising a single population jointly assembled from collagen types I and V. The surface of these fibrils is associated with leucine-rich proteoglycans and other fibril-associated collagens with interrupted triple helices, which help to link and integrate the fibrillar collagen with other components of the ECM (Chen et al., 2015).

While various models have offered different perspectives on corneal transparency, the prevailing framework focuses on a limited set of criteria that influence this property (Spadea et al., 2016): (i) the thickness of the corneal stroma, (ii) the density and diameter of collagen fibrils, (iii) the differential refractive index between fibrils and the surrounding matrix, and (iv) the spatial arrangement of the fibril array.

### 2.5 Descemet’s membrane

Descemet’s Membrane (DM) is a thin layer located at the posterior end of the cornea, primarily composed of laminins, nidogens, perlecan, and type IV collagen. It is divided into a non-banded layer adjacent to the stroma, approximately 0.3 µm thick, followed by an anterior striped zone that is 2–4 µm thick, and a posterior nebulous layer secreted by the neighboring endothelium (Johnson et al., 1982; Levy et al., 1995; Chaon et al., 2016; Price et al., 2021). As a basement membrane, DM is essential for anchoring the endothelium and facilitating the transfer of molecules and nutrients into the stroma. Additionally, DM plays a crucial role in maintaining corneal integrity by serving as an attachment site for corneal endothelial cells (Johnson et al., 1982; Levy et al., 1995; Saikia et al., 2018). Despite its supportive functions, DM has limited regenerative capacity in response to infections and corneal injuries (Marino et al., 2017).

Another recently identified layer, known as the “pre-DM” or “Dua’s layer,” has been reported in the posterior cornea. This well-defined, acellular layer is located just anterior to DM, separating at the level of the last row of stromal keratocytes. Dua’s layer consists of thin lamellae made up of tightly packed collagen bundles (Dua et al., 2013). Despite the initial controversy surrounding the discovery of the Dua’s layer, subsequent studies have provided evidence confirming its presence across different age groups, including pediatric patients. Although some aspects remain under investigation, the growing body of histological and clinical data has substantiated the anatomical relevance of the Dua’s layer in corneal research and surgical practice (Dua et al., 2023).

### 2.6 Corneal endothelium

The endothelium is a single, confluent layer of hexagonal, tightly packed squamous cells with discontinuous apical tight junctions, situated at the posterior of the cornea adjacent to DM (Bourne and Kaufman, 1976; Laing et al., 1976; Blatt et al., 1979). Its primary role is to regulate stromal hydration and facilitate the passage of nutrients from the aqueous humor into the cornea (Hedbys et al., 1963; Mishima, 1968; Mayes and Hodson, 1978).

## 3 Corneal pathologies and their current treatments

Corneal diseases rank as the fifth most common cause of blindness globally. As the outermost layer of the eye, the cornea is highly susceptible to various damaging factors, including mechanical, chemical, and thermal insults (Flaxman et al., 2017). According to a 2022 World Health Organization (WHO) report, 2.2 billion people worldwide experience vision impairment or blindness (www.who.int/news-room/fact-sheets/detail/blindness-and-visual-impairment). To improve transplantation outcomes, conventional full-thickness corneal transplants (penetrating keratoplasties) are increasingly being replaced by lamellar keratoplasties, where only specific layers of the cornea are transplanted, such as in deep anterior lamellar or endothelial keratoplasties (Ghezzi et al., 2015). When allogeneic corneal grafts are not viable, keratoprosthesis—replacement with an artificial cornea—can be employed, although this approach carries a high risk of complications, including glaucoma and infections (Iyer et al., 2018).

Corneal ulceration, a prevalent corneal pathology, typically involves epithelial defects with or without stromal loss, often accompanied by inflammation (Farghali et al., 2021). Rapid deterioration of corneal tissue and stromal necrosis can lead to perforation, resulting in blindness from scarring and astigmatism (Huerva et al., 2014). Corneal ulcers can be caused by bacterial (e.g., *Staphylococcus*, *Streptococcus*, *Pseudomonas* (Lakhundi et al., 2017)), fungal (e.g., *Aspergillus, Fusarium* (Sharma et al., 2022)), viral (e.g., *Herpes simplex type 1 immune-related* (Su et al., 2022)), or traumatic origins (Kang et al., 2020). In advanced cases, the disease often resists high-dose topical antibiotics and systemic corticosteroids (Palioura et al., 2016; Egrilmez and Yildirim-Theveny, 2020). Late-stage interventions include bandage or scleral lenses, amniotic membrane (AM) transplants, conjunctival flaps, autologous serum, tarsorrhaphy, or keratoplasty, depending on the extent of infection or perforation (Tuli et al., 2007; Bremond-Gignac et al., 2019).

Stromal or interstitial keratitis is defined as a nonulcerative and nonsuppurative inflammatory reaction accompanied by neovascularization and cellular penetration in the stroma. Etiologies of interstitial keratitis could be triggered as an immune response to autoimmune diseases (such as Cogan’s syndrome and Hodgkins disease), parasitic diseases (leishmaniasis, malaria, amoebiasis), viral diseases (herpex simplex I and II, influenza, measles), and bacterial diseases (syphilis, tuberculosis keratitis, lyme-associated keratitis, interstitial keratitis) (Schwartz et al., 1998). The primary cause of interstitial keratitis worldwide is syphilis bacterial infection. In the early stages, the disease responds well to high doses of topical steroids (Tu, 2022); however, in some cases, there is a risk of recurrence (Goegebuer et al., 2003; Vingopoulos et al., 2020; Li X. et al., 2023).

Corneal dystrophies, a group of genetic disorders affecting various corneal layers (Weiss et al., 2015, p. 2), include conditions like Fuchs’ endothelial dystrophy. This disease disrupts corneal hydration and can lead to bullous keratopathy, characterized by vision loss and corneal opacity (Kinoshita et al., 2018). Fuchs’ dystrophy progresses through three stages, influenced by mutations in genes like COL8A2, TCF4, and SLC4A11, affecting collagen and endothelial function (Riazuddin et al., 2010; Schmedt et al., 2012). Early management may involve hypertonic saline and bandages, but advanced cases often require corneal transplantation (Mataftsi et al., 2013; Koizumi et al., 2014; Yoshida et al., 2023). Although AM transplantation can alleviate symptoms and improve wound healing (Espana and Birk, 2020; Pires et al., 1999), more clinical trials are needed to assess long-term efficacy (Siu et al., 2014).

Keratoconus, a bilateral ectatic disease, causes progressive thinning and a cone-like protrusion of the cornea, leading to astigmatism and myopia (Claessens et al., 2022). Corneal cross-linking with ultraviolet light and riboflavin is effective in halting disease progression (Pagano et al., 2020; Aytekin and Pehlivan, 2021). Lamellar keratoplasty, which minimizes risks of endothelial rejection, is increasingly preferred over full-thickness keratoplasty for advanced cases (Liu et al., 2015; Feizi et al., 2023).

## 4 Biomaterial-based approaches for corneal tissue engineering

One of the earliest efforts to construct human cornea equivalents was achieved by Griffith et al., who used immortalized human corneal cells without relying on scaffolds or biomaterials (Griffith et al., 2000). Although these corneal equivalents replicated the main physical and physiological functions of the cornea, they presented several limitations. Advances in artificial cornea development highlighted the need to incorporate various scaffolds. Griffith and her team extensively researched crosslinked human collagen as a safe and stable corneal substitute (Li et al., 2003; Suuronen et al., 2004; Liu et al., 2008; Merrett et al., 2008). This research led to clinical trials involving the implantation of biosynthetic human recombinant collagen-based corneas in human patients, which successfully promoted corneal regeneration and restored vision (Fagerholm et al., 2009; 2014). This was a significant milestone in regenerative medicine and corneal tissue engineering.

Numerous investigations have focused on developing hemicorneal models as partial equivalents of the human cornea, designed to replicate specific layers or functions. These models are particularly useful for studying toxicity, drug delivery, and the fundamental biology of the human cornea without animal models. Hemicorneas can be constructed using human primary corneal LESCs and keratocytes anchored by scaffolds such as chitosan, glycosaminoglycan, human KP lenticules, collagen, and laminins (Builles et al., 2007; Barbaro et al., 2009). Innovative tissue-engineered corneas, which combine natural and synthetic biopolymers with primary corneal cells or stem cells, hold promise for future corneal repair applications.

Hydrogel scaffolds have attracted considerable attention in corneal regeneration due to their excellent biocompatibility, viscoelasticity, and water content, making them ideal substitutes for donor or artificial corneas (Vrehen et al., 2023; Wang M. et al., 2023). Biopolymers frequently studied for corneal construction include collagen, gelatin, methacrylated gelatin (GelMA), silk, hyaluronic acid (HA), and decellularized corneal tissues (Table 2). Collagen hydrogels, in particular, have shown promise as scaffolds for delivering LSCs and supporting full epithelial regeneration post-transplantation. Other bioengineered scaffolds, such as fibrin-agarose combined with allogeneic cells, are currently undergoing clinical trials as potential alternatives for ulcerated corneas (González-Andrades et al., 2017). However, the limited mechanical properties of natural polymers often necessitate combining them with synthetic hydrogels to create hybrid scaffolds with enhanced mechanical strength and customizable properties (Vrehen et al., 2023).

**TABLE 2:** Various types of scaffolds utilized in tissue engineering studies for the replacement of different layers of the cornea.

Cornea layer	Scaffold	Reference
Anterior cornea	Gelma/Polyethylene Glycol Diacrylate (PEGDA)	Hamedi et al. (2024)
	Decellularized human cornea	Polisetti et al. (2021)
Epithelium-stroma	Thiolated gelatin-methacrylated HA	Agrawal et al. (2024)
	Gelma-oxidized HA	Wang et al. (2023c)
	HA	Koivusalo et al. (2019), Yazdani et al. (2019)
	Decellularized porcine cornea	Ahearne and Lynch (2015), Wang et al. (2020)
Stroma	GelMa -grid poly (ε-caprolactone)-poly (ethylene glycol) microfibrous scaffold	Fernández-Pérez et al. (2020)
	Poly (ε-caprolactone)	Fernández-Pérez et al. (2020)
	Oligoethylene glycol-dendronized chitosans	Feng et al. (2021)
	Silk fibroin with gelatin	Sahi et al. (2021)
	Nanocellulose-dexamethasone-collagen	Xeroudaki et al. (2023)
	Recombinant collagen-microfibers-gold-nanoparticle- barium titanates	Kong et al. (2024)
	Plastically compressed collagen scaffold	Xiao et al. (2014)
Endothelium	Functionalized Polyethylene glycol (PEG)	Brown et al. (2023)
	Heparin-modified gelatin	Niu et al. (2014)
	Methacrylated gellan gum	Seo et al. (2023)

**FIGURE 2:**
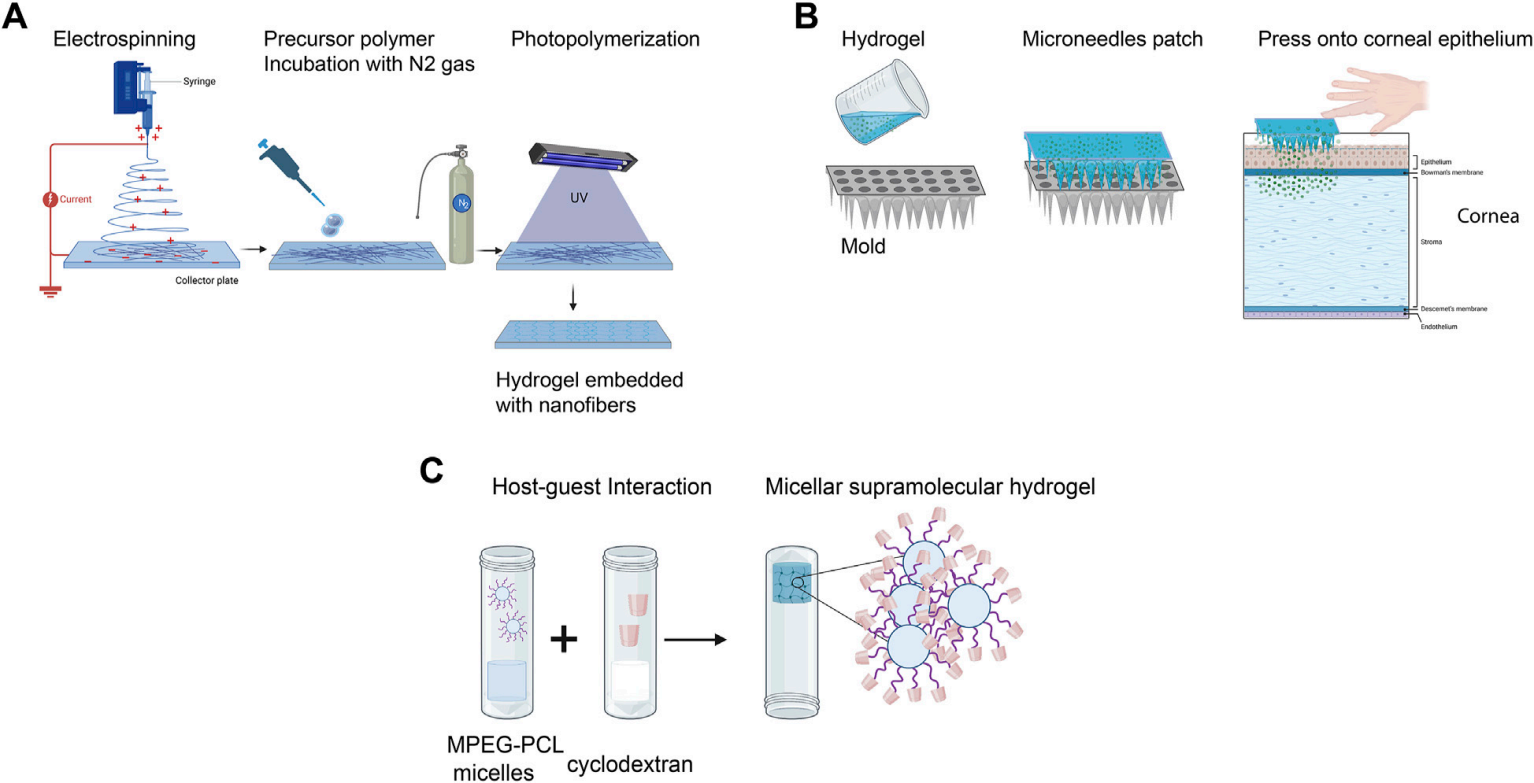
Bioengineered corneal scaffolds. **(A)** Fabrication of nanofibrous corneal scaffold nanofibers by electrospinning. **(B)** Utilization of hydrogel with microneedles patches for enhanced ocular drug delivery. **(C)** Development of micellar supramolecular hydrogel for efficient hydrophobic ocular drug delivery. (Adapted and modified from (Zhang et al., 2016; Arica et al., 2021; Shi et al., 2022; Wang et al., 2024). Figure designed with Biorender (https://app.biorender.com).

Electrospinning has emerged as a valuable technique for creating corneal scaffolds with interconnected nanofibers, typically less than 1,000 nm in diameter, closely mimicking native corneal architecture (Nosrati et al., 2020). Electrospun nanofibers, such as GelMA composite fibers, have demonstrated potential for corneal applications, as shown in a study by Arica et al., where GelMA prepolymer solutions were homogenized and nitrogen-washed to maintain an inert state before casting and photopolymerization (Figure 2A) (Arica et al., 2021). Electrospinning synthetic biocompatible polymers, including polyε-caprolactone, polylactic acid (PLA), and poly (lactic-co-glycolic acid) (PLGA), has also been explored to fabricate aligned scaffolds that support corneal cell proliferation and migration (Fernández-Pérez et al., 2020; Wang M. et al., 2023). Additionally, electrospun silk-gelatin scaffolds have been used to produce cornea analogs with comparable physicochemical properties (Sahi et al., 2021). This combination of electrospinning with natural and synthetic materials offers a promising approach for developing corneal scaffolds with improved mechanical strength and functionality.

In conjunction with these regenerative strategies, integrating advanced drug delivery systems into bioengineered corneal constructs offers significant therapeutic benefits, including reduced inflammation and enhanced tissue healing. One such technology is microneedle injection, which has been widely investigated for efficient drug delivery. A recent study designed a minimally invasive dissolving microneedle array patch using poly D L-lactide and HA (Figure 2B). These acellular patches demonstrated high efficacy in treating fungal keratitis *in vivo* (Shi et al., 2022). Other hydrogels, such as nanowafers, have also been enhanced with nanodrug delivery systems (Yuan et al., 2015).

Recent advancements in micelle-laden hydrogels have shown promising results for treating glaucoma and other ocular disorders. These hydrogels utilize encapsulated PEG-PLA micelles within a triple-crosslinked hydrogel matrix, enabling prolonged and sustained drug release (Zhao et al., 2023). Constructing such micellar supramolecular hydrogels enhances precorneal retention time due to the hydrogel’s physical crosslinking and improves corneal drug penetration facilitated by the micellar structure. Briefly, supramolecular hydrogels are formed by integrating cyclodextrins (CDs) with monomethoxy PEG block polymers, which increase the delivery efficiency of hydrophobic drugs (Figure 2C) (Zhang et al., 2016).

## 5 Cell-based strategies for corneal tissue engineering

Regenerative medicine is emerging as a transformative approach with the potential to replace conventional pharmacological treatments across a wide range of diseases. It involves reviving or rejuvenating human tissues, or even organs, using different types of cells to restore normal function (Mousaei Ghasroldasht et al., 2022). Significant advancements in stem cell therapy have been made recently, with numerous approved and ongoing clinical trials. Commonly utilized cell types in these therapies include adult stem cells, mesenchymal stem cells (MSCs), embryonic stem cells (ESCs), and induced pluripotent stem cells (iPSCs) (Chen et al., 2022).

In corneal treatments, one major application of stem cell therapy is in addressing limbal stem cell deficiency (LSCD). The human limbus contains deep crypts surrounded by the palisades of Vogt, and disruptions in this limbal stem cell (LSC) niche framework lead to significant deficiencies (Ghareeb et al., 2020). LSCD staging is based on the extent of limbal involvement, and detecting precise changes requires advanced imaging tools like *in vivo* confocal microscopy and optical coherence tomography (OCT) (Banayan et al., 2018). Corneal epithelium reconstruction involves isolating and expanding cells from the limbal niche *in vitro* or *ex vivo*. For unilateral disease, a biopsy from the unaffected eye provides cells that are cultured and transplanted back into the patient, followed by localized immunosuppression to prevent immune reactions. This approach has shown significant improvements in corneal integrity for LSCD patients (Kolli et al., 2010; Ramírez et al., 2015).

A recent clinical approach expanded LSCs *ex vivo* under animal-free conditions to reduce patient risks. In this case, autologous LSCs were cultured on human AM under good manufacturing practice conditions, yielding positive results in reversing LSCD (Kolli et al., 2010). However, variations in AM properties limit its consistency, and alternatives with uniform characteristics are sought. Fibrin has been considered, though with lower success rates compared to AM (Rama et al., 2001). Despite success, LSCs alone have limitations in sustaining corneal epithelial homeostasis (Dua et al., 2009).

An alternative to LSCs is using oral mucosa (OM) tissue for patients with LSCD and other ocular surface disorders. OM is rich in stem cells resembling corneal epithelial structures and can form stratified cell layers on the corneal surface. Transplanted OM-derived cells maintain stemness while adopting an epithelial phenotype (Oliva et al., 2020; Gong et al., 2022). Clinical trials have shown that OM-cultured cell sheets improve epithelialization, visual acuity, and transparency in patients with severe ocular disorders (Nishida et al., 2004; Burillon et al., 2012; Kim et al., 2018b). This approach is an autologous-based therapy for corneal disorders (Bandeira et al., 2020). These sheets of autologous cells are treated and expanded *ex vivo* to be subsequently transplanted to the cornea of the patient. Various clinical trials revealed re-epithelialization of patient corneas after autologous OM-cultured epithelial cell sheet transplantation, with significant enhancement of visual strength and transparency. Treatment with OM epithelial transplantation supported by rigid contact lenses is also promising for restoring the vision of patients with severe ocular surface disorders (Sotozono et al., 2020).

Clinical studies of adipose-derived adult stem cells as implants in five patients with keratoconus yielded an optically transparent autologous stromal graft for up to 3 years without any noted complications (El Zarif et al., 2021). Another clinical cell therapy approach was used in a single-group study, which included 11 people with no CECs detected and who were diagnosed with bullous keratopathy. Injection of ROCK inhibitor-supplemented human CECs improved visual acuity and improved CEC density 168 days post-cellular injection (Kinoshita et al., 2018).

Recent efforts in cell-based therapies focus on autologous approaches using adult stem cells or iPSCs. Human adipose-derived stem cells (ADSCs) can differentiate into epithelial-like cells for autologous therapies. For instance, ADSCs implanted in patients with keratoconus produced transparent grafts, maintaining functionality for up to 3 years (El Zarif et al., 2021).

Other approaches, such as the use of corneal stromal stem cells (CSCs), restore transparency in models with induced scarring (Du et al., 2009). This population of cells has been successfully characterized as an individual population showing essential qualities of adult stem cells; these cells are localized just below the limbus basal membrane (Funderburgh et al., 2016). Although CSCs offer essential regenerative qualities, their scarcity and the challenge of harvesting limit their use. Moreover, achieving high numbers of CSCs by *ex vivo* subculturing and proliferation could be challenging (Yusoff et al., 2022). ADSCs, by contrast, are readily available, and transplantation trials have shown sustained transparency in animal models (Arnalich-Montiel et al., 2008). Additionally, proof-of-concept trials have also validated the use of bone marrow-derived MSCs transplantation as a safe and effective procedure, with results similar to those of *ex vivo* cultured allogenic LSC transplantation (Calonge et al., 2019). To sum up, it is much easier to harvest and culture CSCs (namely, limbal stromal MSCs) than the epithelial phenotype stem cells in this region. Furthermore, since these cells are mesenchymal in origin (lacking MHC type 2), they are non-immunogenic and can even be harvested and cultured from the limbal region of donor corneal tissue harvested from cadavers.

Cell-based approaches that are scaffold-free, have been also employed in cornea tissue engineering, these are techniques using cells in suspension, spheroid aggregates forms, or even as sheets (Shah et al., 2008). To date, many fabrication techniques that employ both advanced scaffold-based and scaffold-free methods for the repair of damaged cornea to native structure have been developed (Guérin et al., 2021). The self-assembly process of scaffold-free approaches, enable the formation of stable structures via cell-to-cell interactions, relying on non-covalent interactions to organize molecules (Athanasiou et al., 2013; Nune et al., 2013).

Corneal organoids, 3D models of corneal tissue, show potential in developmental studies, disease modeling, and possibly as replacement organs (Lancaster and Knoblich, 2014). These self-organizing organoids (Meyer-Blazejewska et al., 2010; Nakano et al., 2012; Susaimanickam et al., 2017; Wan et al., 2024) can derive from primary cells, adult stem cells, ESCs, or iPSCs, and are influenced by external factors such as growth factors and ECM substrates (Clevers, 2016).

The corneal organoids replicate the primary cellular components of the cornea in a stable system, offering a platform to study long-term cellular phenotypes associated with diseases such as dry eye disease, as well as developmental processes. These models facilitate the exploration of interactions between various cell types and the ECM (Foster et al., 2017). Notably, corneal organoid cultures have been shown to preserve the limbal epithelial niche for up to 1 month. Epithelial sheets that retain the limbal epithelial phenotype have been successfully engineered from a single organoid. Furthermore, intact organoids have been successfully engrafted onto the limbus in a rabbit model, underscoring their potential for treating ocular surface diseases associated with limbal stem cell deficiency (LSCD) (Higa et al., 2020). Corneal organoids also hold significant promise for applications in drug screening, gene editing, and tissue replacement (Foster et al., 2017). Additionally, these organoids can be used in cell therapy as living biobanks and have high potential in the development of gene therapies, optogenetics, and as a source of transplantable cells (Zhong et al., 2014; Susaimanickam et al., 2017; Chichagova et al., 2020; Lu and Le, 2024).

However, a major challenge in organoid development is maintaining proliferative capacity, which limits their usability in extended testing (Lancaster and Huch, 2019). Other limitations include metabolic build-up, reduced glucose and oxygen diffusion, incomplete maturation, eventual cell necrosis, and a decline in cell proliferation over time (Andrews and Kriegstein, 2022).

## 6 Bioprinting as a bioengineering tool for corneal tissue

Bioprinting is an advanced bioengineering technique characterized by the additive, layer-by-layer deposition of cells within biomaterials or bioinks into spatially interconnected and defined structures using automated 3D technologies (Wang et al., 2022b). These structures are typically designed using computer-aided design (CAD) models or generated through magnetic resonance imaging or computed tomography (Shelmerdine et al., 2018; Schmieg et al., 2022).

Bioinks are fluidic biomaterials used in bioprinting to incorporate living cells, either in suspension or aggregate form (Zhang et al., 2018). When selecting a bioink, key factors to consider include printability, biocompatibility, biodegradability, mechanical stability, integrity, and similarity to the native composition (Wang et al., 2022b).

An emerging and promising technology in this field is Freeform Reversible Embedding of Suspended Hydrogels (FRESH). This technique provides mechanical support through a thermoreversible gelatin slurry bath, creating an environment conducive to cellular migration and interaction (Corbett et al., 2019).

**FIGURE 3:**
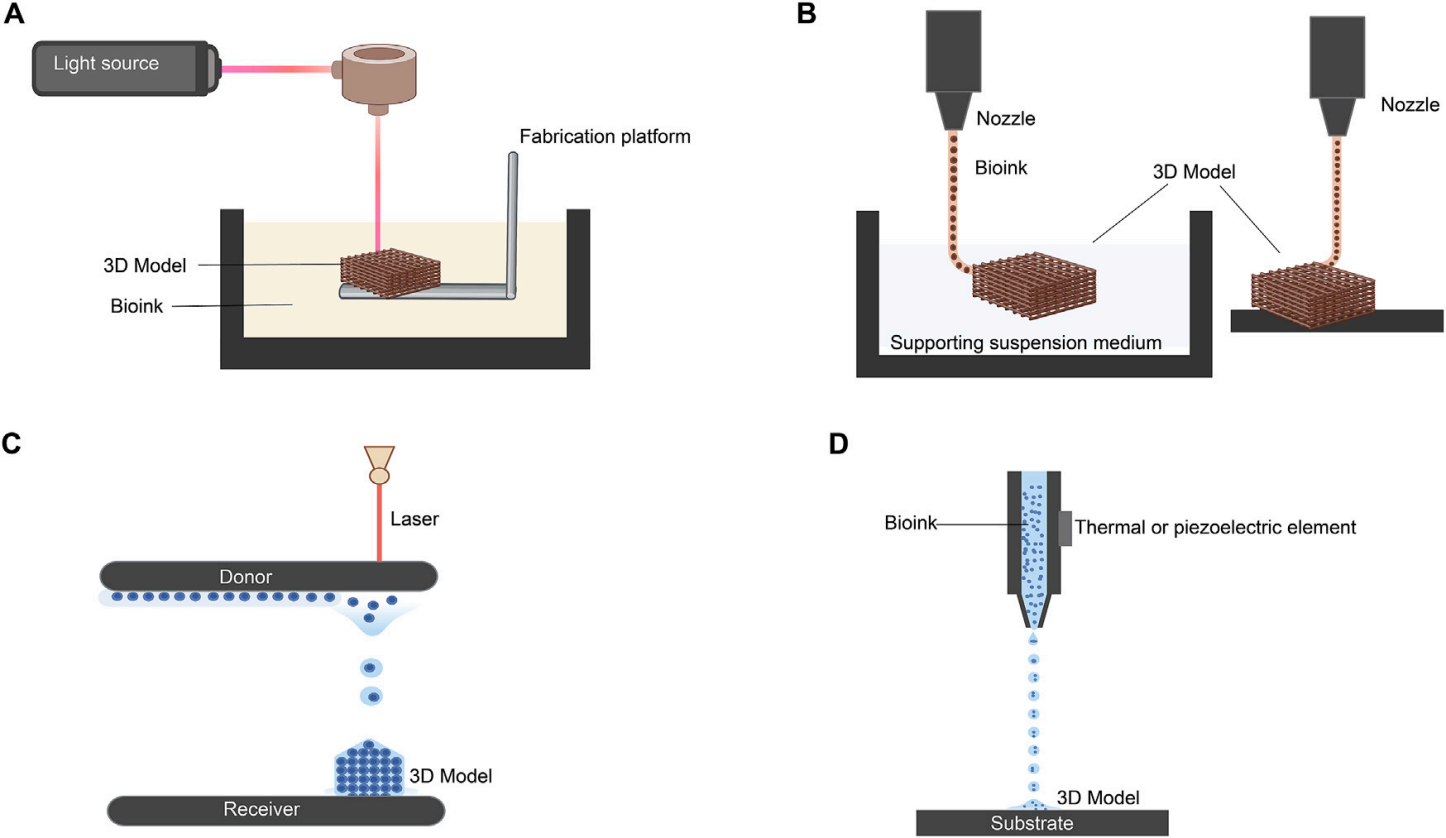
Most common strategies for 3D bioprinting. **(A)** Stereolithography-based 3D bioprinting. **(B)** Extrusion-based methods using suspended and direct extrusion bioprinting. **(C)** Laser-assisted bioprinting. **(D)** Inkjet bioprinting via piezoelectric and thermal elements. (Adapted and modified from (Cooke and Rosenzweig, 2021; Kumar et al., 2021; Yang et al., 2021; Li W. et al., 2023). Figure designed with Biorender (https://app.biorender.com).

The primary strategies employed in 3D bioprinting include stereolithography, extrusion, laser-assisted, and inkjet bioprinting (Figure 3) (Matai et al., 2020). Stereolithography bioprinting is based on crosslinking a liquid photocrosslinkable bioink upon exposure to a specific light source, controlled by CAD, to produce a 3D model (Figure 3A) (Li W. et al., 2023). Extrusion-based bioprinting, the most common approach for corneal applications, creates structures through continuous deposition (Figure 3B) (Cooke and Rosenzweig, 2021). Laser-assisted bioprinting uses a focused laser beam to transfer a precise volume of bioink from a donor substrate to a receiver (Figure 3C) (Yang et al., 2021). Finally, inkjet bioprinting employs thermal or piezoelectric mechanisms to eject bioink droplets from the printhead, forming layer-by-layer structures (Figure 3D) (Kumar et al., 2021).

### 6.1 Bioprinting of the corneal epithelium

Limbal epithelial stem cells demonstrate strong proliferative and migratory capabilities, allowing them to migrate from the limbus to the epithelial layer, where they differentiate into epithelial cells (Wang B. et al., 2023). This suggests the hypothetical possibility of forming the epithelial layer without the need for bioprinting. Bioengineered cell sheets have become the primary approach for generating a functional corneal epithelial layer *in vitro* (Bardag-Gorce et al., 2013; Kobayashi et al., 2013; Venugopal et al., 2020). Consequently, compared to research on the corneal stroma, far fewer studies have focused on 3D bioprinting for the corneal epithelium (Table 3).

**TABLE 3:** Summary of recent significant studies on 3D bioprinting of corneal epithelium: a comparative analysis of bioprinting parameters, cell sources, technical approaches, bioinks, and key findings.

Cell source	Bioprinting technique	Bioink	Biological analysis post bioprinting	Cell concentration	Nozzle diameter	References
Human CEC	Extrusion	Collagen/gelatin/alginate hydrogel	Cytocompatibility Proliferation and epithelial markers	10^6^ cells/mL	—	Wu et al. (2016)
	DLP and 3D extrusion	Alginate/gelatin	Cytocompatibility	—	250 µm	Zhang et al. (2019)
Human ESC-LESC	Laser-assisted	Laminin 521/HA Collagen I hydrogel	Cytocompatibility Limbal epithelial markers	30*10^6^ cells/mL	—	Sorkio et al. (2018)
Rabbit LESC	DLP	GelMA HA glycidyl methacrylate	Cytocompatibility Proliferation and epithelial markers	2*10^7^ cells/mL	—	Zhong et al. (2021)

### 6.2 Bioprinting the corneal stroma

Numerous studies on corneal stroma bioprinting have been conducted using various bioprinting techniques, 3D software designs, and cell types (Table 4).

**TABLE 4:** Summary of recent significant studies on 3D bioprinting of corneal stroma: a comparative analysis of bioprinting parameters, cell sources, technical approaches, bioinks, and key findings.

Cell source	Bioprinting Technique	Bioink	Biological analysis post bioprinting	Cell concentration	Nozzle diameter	References
Human keratocytes	Extrusion	Alginate/Gelatin/Collagen	Cytocompatibility ECM proteins expression	3*10^6^ cells/mL	22G	Kutlehria et al. (2020)
		Collagen I	Cytocompatibility	10^5^ cells/mL	25G	Song et al. (2022)
		GelMA	Cytocompatibility MMP activity ECM synthesis	10^6^ cells/mL	260 µm	Bektas and Hasirci (2019)
		Gelatin Polyethylene glycol (PEG)	Cytocompatibility Proliferation Keratocyte marker	3*10^6^ cells/mL	838 µm	Hull et al. (2021)
	Stereo-lithography	GelMA	Cytocompatibility Keratocyte markers	8*10^6^ cells/mL	—	Mahdavi et al. (2020b)
	Drop-on-demand (DoD)	Collagen Collagen/Agarose	Cytocompatibility Keratocyte markers	10 6 cells/mL	300 µm	Duarte Campos et al. (2019)
Human corneal fibroblasts	DLP	Decellularized cornea/GelMA	Cytocompatibility ECM and keratocyte markers *In vivo* healing rate	10^5^ cells/mL	—	Zhang et al. (2023)
Rabbit cornea stromal cells	Extrusion	HA-GelMA	Cytocompatibility Proliferation ECM and stromal markers	10^6^ cells/mL	300 μm	Wang et al. (2022a)
Human cornea stromal cells	Extrusion	Cellulose/Alginate/Col I	Cytocompatibility ECM markers *Ex vivo* expansion	1.5*10^6^ cells/mL	25/27 G	Boix-Lemonche et al. (2023)
Human Adipose stem cells	Laser-assisted	Laminin 521/HA Collagen I	Cytocompatibility Proliferation Key protein expression	30*10^6^ cells/mL	—	Sorkio et al. (2018)
	Extrusion	HA/collagen	Cytocompatibility Innervation Stromal markers *Ex vivo* integration	10*10^6^ cells/mL	100 µm	Mörö et al. (2022)

Corneal stroma bioprinting primarily utilizes collagen bioink due to its biocompatibility (Osidak et al., 2020). However, to address the weak mechanical properties of collagen, it is often combined with other bioinks, such as agarose (Duarte Campos et al., 2019). Duarte Campos et al. employed the Drop-on-Demand (DoD) technique to achieve a corneal stroma structure while maintaining transparency (Duarte Campos et al., 2019). A limitation of this study is that the proposed bioprinted stroma lacks the organized collagen fibril architecture found in the native cornea, which impacts the biomechanical stability of the model.

In the work of Isaacson et al., corneal stroma was bioprinted using extrusion bioprinting with primary keratocytes embedded in alginate and collagen (Isaacson et al., 2018). However, the assessment of biological compatibility was limited to LIVE/DEAD viability assays and trypan blue staining. Additionally, the round morphology of bioprinted keratocytes post-printing indicates a lack of differentiation within the hydrogel.

Due to its favorable mechanical properties and functionality, GelMA has become widely used in bioprinting. Constructs bioprinted with GelMA loaded with corneal keratocytes showed high transparency and cell viability for up to 1 week, although cell proliferation declined significantly afterward (Bektas and Hasirci, 2019). Visible light-based stereolithography using GelMA also demonstrated optimal transparency immediately after bioprinting. Embedded corneal keratocytes retained high gene expression of specific markers and exhibited good viability 7 days post-bioprinting (Mahdavi et al., 2020a).

A team from Stanford and Carnegie Mellon Universities formulated and stabilized a variety of polymers to use as UNION bioinks. High stromal cell viability was observed 24 h post-printing, and the cell phenotype was maintained for 7 days using gelatin as a bioink (Hull et al., 2021). Recently, HA-based bioinks were developed for laser-assisted bioprinting, demonstrating promising physicochemical properties and high cellular compatibility (Sorkio et al., 2018). However, there has been no validation of corneal keratocyte phenotype or integration post-bioprinting. Other studies have achieved cell-laden structures with enhanced mechanical properties using HA bioink (Mörö et al., 2022).

### 6.3 Bioprinting of the corneal endothelium

Very few studies have attempted to bioprint corneal endothelial layers using solely extrusion-based bioprinters (Table 5). In parallel, the most effective approaches in corneal endothelial therapy currently involve artificial ROCK inhibitors, Descemet stripping only, and cellular and gene therapy techniques (Rocha-de-Lossada et al., 2021).

**TABLE 5:** Summary of recent significant studies on 3D bioprinting of corneal endothelium: a comparative analysis of bioprinting parameters, cell sources, technical approaches, bioinks, and key findings.

Cell source	Bioprinting technique	Bioink	Biological analysis post bioprinting	Cell concentration	Nozzle diameter	References
Human corneal endothelial cells	Extrusion	Gelatin-based	mRNA expression in rabbits Tight junction and adhesion proteins Na^+^–K^+^ ATPase	—	—	Kim et al. (2018a)
Human PSC-corneal endothelial-like cells		HA/Collagen IV/ Laminin 521	Cytocompatibility Tight junction and adhesion proteins Na^+^–K^+^ ATPase	5.15–106 cells/mL	100 µm	Grönroos et al. (2024)

In recent studies, researchers have employed 3D extrusion bioprinting to deposit human corneal endothelial cells modified with RNase 5 plasmid vectors. This modification significantly enhanced cell survival and proliferation by activating cyclin and inhibiting apoptotic protein 4. These modified cells were bioprinted onto lyophilized AM and grafted into rabbit models, leading to notable improvements in edema and corneal thickness (Kim et al., 2018b). This approach underscores the potential of bioengineered 3D bioprinted corneal endothelial models for transplantation.

Additionally, extrusion-based 3D bioprinting has been used to develop integrated tissue from hiPSC-derived corneal endothelial cells, which exhibited essential endothelial characteristics and maintained cell viability for over 1 week. This was achieved using a specialized bioink composed of laminin, collagen IV, and HA (Grönroos et al., 2024).

## 7 Cell-free approaches for corneal tissue engineering

**FIGURE 4:**
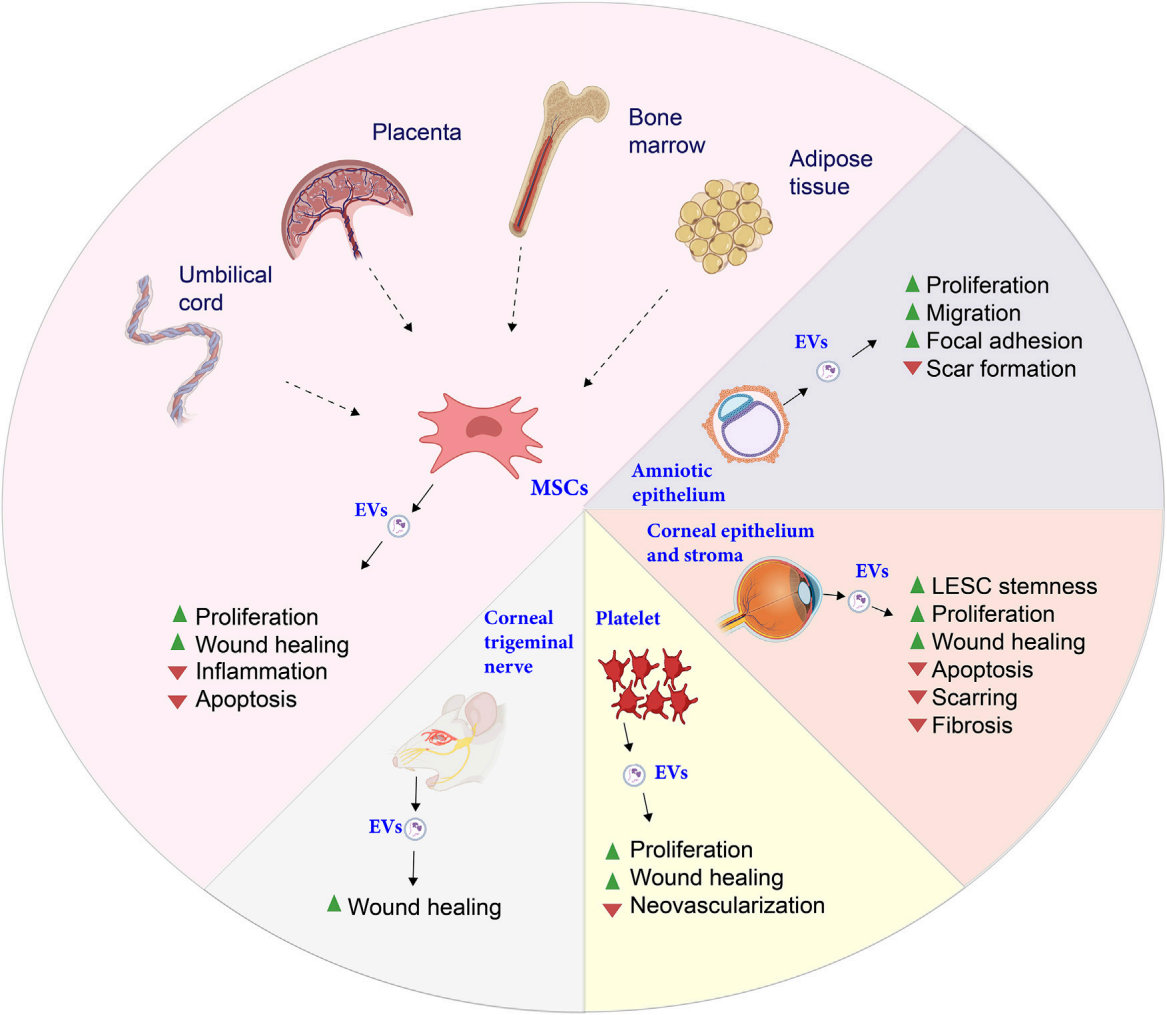
Summary of the known functions of extracellular vesicles (EVs) according to their origin. While most of them act positively on cell proliferation, each type of EVs modulate differently secondary cellular and physiological processes. Figure designed with Biorender (https://app.biorender.com).

The use of stem cells as therapeutic agents has raised clinical safety concerns due to their heterogeneity and the potential for immune responses upon transplantation (Dua et al., 2009; Shi et al., 2012; Masterson et al., 2021; Oh et al., 2021). As a result, cell-free approaches are gaining traction in research, aiming to induce “cell homing” and recruit tissue-resident cells through bioactive molecules (Thalakiriyawa and Dissanayaka, 2024). Among these molecules are secretomes—such as cytokines, extracellular vesicles (EVs), and growth factors—produced by stem cells. EVs from various cellular sources have shown considerable promise in corneal repair and regeneration (Figure 4).

Research on EVs derived from CSCs has demonstrated their regenerative and healing properties, including scar reduction, suppression of fibrosis markers, and enhancement of LESC properties via the Notch pathway (Shojaati et al., 2019; Santra et al., 2022; Wang et al., 2023e; Santra et al., 2024). EVs from human placental mesenchymal stem cells (MSCs) have also been found to enhance cell proliferation, exhibit anti-inflammatory effects, and facilitate wound healing in CECs (Tao et al., 2019). Similarly, EVs from bone marrow-derived MSCs promote corneal repair, protect endothelial and epithelial cells from apoptosis via caspase-3 inhibition, and support healing processes (Buono et al., 2021; Nuzzi et al., 2021; Saccu et al., 2022; Tati et al., 2024).

Moreover, EVs from placental and umbilical cord MSCs aid in wound healing by reducing inflammation and accelerating corneal repair (Tao et al., 2019; Liu et al., 2022). EVs from ADSCs are known to reduce inflammation in dry eye disease and support epithelial repair in diabetic models (Yu et al., 2020; Wang et al., 2023d; Wang et al., 2023 G.). EVs from other sources also contribute uniquely to corneal healing. For instance, EVs from corneal myofibroblasts enhance endothelial cell migration and proliferation (Yeung et al., 2022), while those from human amniotic epithelial cells stimulate epithelial and stromal cell proliferation, reduce scarring, and activate the focal adhesion pathway (Hu et al., 2022; Yi et al., 2024).

EVs are now being utilized within electrospun scaffolds, marking a significant advancement in cell-free regenerative strategies for corneal repair. Electrospun nanofibrous scaffolds, particularly those composed of PLGA, have proven to be highly effective for the delivery of bioactive molecules. Recent studies have demonstrated that these scaffolds, when loaded with MSC-derived EVs, exhibit considerable therapeutic efficacy. In models of corneal and retinal injuries, PLGA nanofibrous scaffolds incorporating MSC-EVs have been shown to significantly enhance tissue regeneration, while reducing fibrosis and scar formation, suggesting their potential for ocular surface reconstruction (Wang et al., 2024).

This emerging approach is not limited to synthetic polymers; EVs have also been integrated into biological scaffolds, such as porcine-derived decellularized ECM and modified nanofibrous structures, further demonstrating their versatility in corneal tissue engineering (Hernandez et al., 2018). Additionally, while electrospun scaffolds have shown promising results, EVs have been incorporated into other biomaterials, such as MSC-EV-loaded chitosan hydrogels, which have similarly facilitated epithelial and stromal regeneration and reduced post-injury scar formation (Tang et al., 2022). The use of EVs within electrospun scaffolds and other biomaterial platforms represents a highly promising avenue in the development of advanced, cell-free therapies for corneal repair, offering both structural support and bioactivity necessary for effective tissue regeneration.

Despite these promising developments, EV-based therapies are still in their early stages, necessitating further research to fully understand their mechanisms of action and to comprehensively characterize EVs for use in corneal regeneration.

## 8 Overcoming current limitations

Despite the broad range of current treatments for corneal pathologies, sight-threatening conditions that require corneal grafts remain challenging to cure due to the limited availability of donor tissues. The risks associated with autologous tissue use and the lifelong immunosuppression required for allogenic grafts make bioengineered corneal tissue an appealing alternative. While emerging treatments, such as stem cell therapy and autologous oral mucosa transplantation, show promise, this discussion focuses on the essential features of a bioengineered cornea.

A key aspect of bioengineered corneas is sourcing cells. To reduce rejection risk, the cells should ideally originate from the patient. It is anticipated that two primary cell sources will be prominent in the coming years: oral mucosa cells and reprogrammed human iPSCs. While oral mucosa cells may lack the ability to transdifferentiate into keratocytes or endothelial cells, iPSCs present risks of genomic instability and require careful monitoring to ensure safety in bioengineered corneas. Furthermore, EVs have emerged as important components in cornea tissue engineering, facilitating cell communication, migration, and promoting tissue regeneration through their diverse cargo (proteins, RNA) (Hutcheon et al., 2019; McKay et al., 2019; 2020). However, no EV-based therapies have received FDA approval for therapeutic use up to this date (Cheng and Kalluri, 2023). As a relatively new field, current EV isolation techniques hold various limitations, including low yield, limited purity, sample heterogeneity, which are particularly problematic for producing clinical-grade EVs (Allelein et al., 2021). Additional challenges appear in direct incorporation of EVs into hydrogels or any type of scaffold due to variability in their loading efficiency between different batches. This issue could be resolved by employing the co-printing method outlined in Bari et al., where EVs are encapsulated within a hydrogel, providing a controlled and sustained release (Bari et al., 2021). There has been few research on loading therapeutic cargo onto EVs to treat occular diseases. Therefore, further research is essential to develop novel EV-related therapeutic strategies in ophthalmology.

As the field progresses, innovative solutions related to hydrogel composition, optimal cell sources, and clinically applicable methods will likely enhance clinical ophthalmology, particularly in cell therapy, organ bioprinting, and personalized medicine. Scaffold-free cell injection strategy might be promising due to the reduced inflammatory response and biomimetic properties. However, implementing this technique requires adherence to strict quality standards, alongside thorough *in vivo* studies, to effectively regulate complex matrix alignment of the cornea (Gouveia et al., 2017a; Syed-Picard et al., 2018). Moreover, cell injection alone is insufficient for regenerating severely damaged cornea, as it lacks the structural matrix support essential for proper cell localization and integration. For instance, injecting stromal cells into the corneal stroma can distort the native fibrillar architecture of the tissue (Alió del Barrio and Alió, 2018; Alió et al., 2019). While transparency and cell organization can be achieved, corneal models generated by scaffold-free approaches often lack long term stability, and mechanical robustness, which is crucial for withstanding the natural pressures of the eye (Guérin et al., 2021).

To achieve an optimal bioengineered cornea, it is essential to replicate the physiological properties of the native cornea and maintain a suitable microenvironment by utilizing suitable scaffolds. Hydration balance is a critical factor, with the native cornea maintaining a hydration limit of approximately 80% (Rinawati et al., 2018). Disruption of this balance characterizes various corneal diseases, including corneal edema (Yao et al., 2020).

The current research on scaffolds requires more focus on the nano-structural properties, cell-scaffold interaction, and scaffold degradation. Recent innovations such as implementation of curved templates has successfully facilitated the production of more accurate corneal curvatures while promoting the alignment of corneal keratocytes and collagen fibrils (Gouveia et al., 2017b). Additionally, Gouveia et al. developed self-lifting analogous tissue equivalents by using peptide amphiphile-coated surfaces with varying anisotropies (Gouveia et al., 2017a). Electrospun polyvinyl acetate and collagen scaffolds were also shown to mimic the cornea’s native structure. Although the scaffolds promoted cell adhesion and proliferation, poor mechanical properties, particularly inadequate strength for surgical suturing, limited their clinical application (Wu et al., 2018).

In bioengineered corneas, scaffold production consistency and scalability also present significant challenges once scaled up for larger clinical trials. Pore size and distribution within scaffolds are additional factors critical to the controlled release of therapeutic molecules. Pore characteristics also alter the scaffold’s rheological properties and mechanical stability, which are essential for supporting cellular activities and diffusion processes within the bioengineered scaffold (Fischer et al., 2019; Hady et al., 2022). Other issues also exist, and are related to the potential risk of inducing immune responses, heterogeneous cell distribution, and achieving a degradation rate that aligns with the cornea tissue formation (Ovsianikov et al., 2018). Polymers including PGA and PLGA are among the most commonly used in tissue-engineered corneas, and have been approved by the US FDA for clinical use. (Hu et al., 2004; Zhang et al., 2008; Deshpande et al., 2013; Arabpour et al., 2019). Additionally, acellular porcine corneal products gained approval of the FDA in 2015 (Zhang et al., 2015; Tran and Hou, 2023).

Even with the extensive research on biomaterials for corneal tissue regeneration, there is still no single agreement on the ideal scaffold or cell source. Clinical trials have yielded limited success and insufficient remodeling during scaffold degradation. While a significant portion of the trials focuses on evaluating various bio-based scaffolds, the majority of registered studies are dedicated to stem cell therapy and bioactive molecules. This highlights the prevailing interest in these two areas within the field of cornea regenerative medicine (Boroumand et al., 2024). Cornea reconstruction using synergism of scaffold-free and scaffold-based biomimetic approaches could advance scaffold functionalities, specifically for corneal stromal reconstruction (Jakab et al., 2010; Ovsianikov et al., 2018; Zurina et al., 2020). Mechanical stability, transparency, and integration with the host tissues, remain critical barriers to successful corneal tissue engineering, in both scaffold-free and biomaterial-based approaches. Thus, translational application is dependent on strategies that can support cell expansion, mimic the cornea ECM composition, and facilitates the overall cornea reconstruction process of a functional tissue.

While 3D bioprinting technology enables precise cellular placement within scaffolds, many current corneal bioprinting studies lack micropatterned cellular arrangements that closely mimic natural tissue. As a result, despite attempts to spatially arrange bioprinted cells, these studies have struggled to replicate the functionality and cellular diversity of the native human cornea (Brassard et al., 2021). Additional considerations, such as stiffness and mechanical properties, are crucial to enable successful suturing or clipping during transplantation (Fuest et al., 2020). Navigating these barriers necessitates a careful recapitulation of embryonic development and organization, allowing cellular self-assembly through patterned gene expression (Warmflash et al., 2014). This kinetic and spatial coordination is key for successful tissue generation and cellular turnover, as adult corneal homeostasis relies on the replication dynamics and spatial balance of corneal stem cells (Strinkovsky et al., 2021). Achieving a precise macroscale structure is influenced by microscale interactions that facilitate the self-organization and assembly of cells and tissues. Bioprinting and cell-specific aggregation are effective strategies for regulating the bottom-up assembly of tissues with complex biological characteristics (Brassard and Lutolf, 2019). A specific challenge in corneal tissue-engineered constructs is maintaining the orthogonal collagen fibrillary microarchitecture which is essential for corneal transparency. Addressing this issue may be possible by utilizing a technique initially developed for cartilage tissue engineering. This approach incorporates 4D bioprinting to remodel collagen fibers using magnetic streptavidin-coated iron nanoparticles, enabling real-time magnetic orientation during the printing process (Betsch et al., 2018).

Given the complexity of the cornea, developing an approved model would require combining advanced bioprinting technologies with other methods, including organoids and stem cells (Brassard and Lutolf, 2019; Jia et al., 2023). Moreover, creating a synergistic blend of 3D bioprinting and organoids would shift the focus to the targeted tissue’s structure and function, enabling the development of deliberate spatial designs that promote higher accuracy and faster organoid self-assembly (Ren et al., 2021). This integrated approach could enhance the production of functional, self-organized artificial tissues or organs. Recent studies have applied this concept to create promising centimeter-scale tissues that exhibit diverse, self-organized features of bioprinted organs, such as the heart, kidney, and intestines (Brassard et al., 2021; Humphreys, 2021; Lawlor et al., 2021).

Another important aspect of tissue engineering involves extensive animal model studies before clinical application. Anatomical studies of various species are crucial for designing engineered tissue models that are applicable to humans (Mukherjee et al., 2022). Selecting the ideal animal model requires an understanding of anatomical and pathophysiological similarities to humans to ensure that the model is fit for purpose (Liebschner, 2004; Ribitsch et al., 2020). Studies on corneas from different organisms help identify models with anatomy closest to the human cornea. Advances in human corneal surgery have often built on veterinary research; for example, studies on dog corneal ultrastructure have contributed to human surgical techniques (Kafarnik et al., 2023). Similarly, in tissue engineering, acellular scaffolds from bovine and porcine corneas have been used to support cellular function in bioengineered models. Extrapolating insights from animal studies is essential for optimizing tissue engineering strategies for human application (Ponce Márquez et al., 2009; Isidan et al., 2019). Collectively, these insights are vital for developing bioengineered corneas that can be used clinically.

Consequently, existing models have not advanced to clinical trials. Moving toward clinical trials would require substantial efforts to develop higher-performing models and a deeper understanding of corneal physiology and anatomy. Addressing issues related to host immune responses to bioartificial corneas and ensuring long-term compatibility is essential for this transition.
